# Case Report: Biallelic Loss of Function ATM due to Pathogenic Synonymous and Novel Deep Intronic Variant c.1803-270T > G Identified by Genome Sequencing in a Child With Ataxia–Telangiectasia

**DOI:** 10.3389/fgene.2022.815210

**Published:** 2022-01-25

**Authors:** Tatiana Maroilley, Nicola A. M. Wright, Catherine Diao, Linda MacLaren, Gerald Pfeffer, Justyna R. Sarna, Ping Yee Billie Au, Maja Tarailo-Graovac

**Affiliations:** ^1^ Department of Biochemistry and Molecular Biology, Cumming School of Medicine, University of Calgary, Calgary, AB, Canada; ^2^ Department of Medical Genetics, Cumming School of Medicine, University of Calgary, Calgary, AB, Canada; ^3^ Alberta Children’s Hospital Research Institute, University of Calgary, Calgary, AB, Canada; ^4^ Section of Pediatric Hematology-Immunology, Department of Pediatrics, Alberta Children’s Hospital, University of Calgary, Calgary, AB, Canada; ^5^ Department of Clinical Neurosciences and Hotchkiss Brain Institute, University of Calgary, Calgary, AB, Canada

**Keywords:** ATM, deep intronic variant, synonymous variant, whole-genome sequencing, splicing, missing heritability, case report

## Abstract

Ataxia–telangiectasia (AT) is a complex neurodegenerative disease with an increased risk for bone marrow failure and malignancy. AT is caused by biallelic loss of function variants in *ATM*, which encodes a phosphatidylinositol 3-kinase that responds to DNA damage. Herein, we report a child with progressive ataxia, chorea, and genome instability, highly suggestive of AT. The clinical ataxia gene panel identified a maternal heterozygous synonymous variant (NM_000051.3: c.2250G > A), previously described to result in exon 14 skipping. Subsequently, trio genome sequencing led to the identification of a novel deep intronic variant [NG_009830.1(NM_000051.3): c.1803-270T > G] inherited from the father. Transcript analyses revealed that c.1803-270T > G results in aberrant inclusion of 56 base pairs of intron 11. *In silico* tests predicted a premature stop codon as a consequence, suggesting non-functional ATM; and DNA repair analyses confirmed functional loss of ATM. Our findings highlight the power of genome sequencing, considering deep intronic variants in undiagnosed rare disease patients.

## Introduction

The ataxia–telangiectasia mutated (*ATM*) gene encodes phosphatidylinositol 3-prime (PI-3) kinase, a 3,056-amino acid protein (Savitsky et al., 1995) that is involved in some critical cellular processes, such as DNA damage repair, apoptosis, cell cycle, metabolism, oxidative stress, and proliferation. Biallelic loss of function variants in *ATM* causes ataxia–telangiectasia (AT; MIM# 208900), a rare autosomal recessive monogenic disease with a prevalence of 1 in 40,000 to 1 in 333,000 live births (https://rarediseases.org/). Ataxia–telangiectasia is a complex, pleiotropic, and neurodegenerative disease characterized by progressive ataxia and movement disorder (chorea and dystonia), telangiectasias, immune defects, radiation sensitivity, chromosomal instability (CIN), and increased risk of malignancy, in particular malignancies of the lymphatic and hematopoietic systems (e.g., lymphomas and leukemia) and the brain (Savitsky et al., 1995). In addition to AT patients with biallelic variants, germline carriers of one copy of the altered *ATM* have a several-fold higher predisposition to cancer than the general population (e.g., breast cancer; MIM#114480) (Swift et al., 1991). Of note, many individuals develop oculocutaneous telangiectasia, but non-classical presentations without telangiectasia and with and without neurological/immune manifestations are also possible. Therefore, ATM syndrome has been proposed as a more appropriate name (Teive et al., 2015).

The *ATM* (NM_000051) gene consists of 63 exons with a transcription start site (ATG) in exon 2 (Gatti and Perlman, 1993). ClinVar (accessed on September 29, 2021) reports more than 1,000 clinically relevant variants in the *ATM* (1,485 classified as pathogenic) (Landrum et al., 2018). Most of the ClinVar pathogenic *ATM* variants result in the absence/truncation of the ATM (Gatti and Perlman, 1993) and involve a) frameshift variants [insertions (237) and deletions (472)] and b) base substitutions (470) that either result in premature termination of the transcript (i.e., nonsense variants; 367) or altered messenger RNA (mRNA) splicing (i.e., splice site donor/acceptor variants; 68) (Landrum et al., 2018). The vast majority of the ClinVar reported pathogenic or likely pathogenic splice site variants, affect the canonical donor/acceptor sites of the NM_000051 transcript (Landrum et al., 2018). However, non-canonical splice variants are uncovered as well. For example, there are variants annotated as synonymous located at the exon/intron boundaries that were confirmed to affect mRNA splicing [c.2250G > A (Teraoka et al., 1999); c.3576G > A (Demuth et al., 2011); c.7788G > A (Broeks et al., 1998)]. Moreover, there are deep intronic variants located hundreds of bases away from the exon/intron boundaries [c.2639-384A > G (Nakamura et al., 2012); c.2839-579_2839-576delAAGT (Pagani et al., 2002); c.3994-159A > G (Coutinho et al., 2005); c.5763-1050A > G (McConville et al., 1996)]. These deep intronic variants were shown to affect the transcripts predominantly by introducing cryptic splice sites and including part of an intron as a pseudo-exon leading to a truncated transcript and RNA decay (Coutinho et al., 2005; McConville et al., 1996; Nakamura et al., 2012; Pagani et al., 2002). These types of non-canonical variants that disrupt mRNA splicing are challenging to detect using standard clinical approaches and may represent an important source of missing heritability (Maroilley and Tarailo-Graovac, 2019) in rare disease patients.

## Case Description

Herein, we report a child with progressive ataxia and chorea for whom clinical ataxia gene panel testing (GeneDx) was inconclusive. The proband, now a five-year-old male, was born at ∼29 weeks to a healthy nulliparous mother and father *via* emergency C-section with Apgar scores of 9 and 9 (1 and 5 min) and birth weight of 1,000 g. The proband was discharged at 34-35 weeks once gastrointestinal food sensitivity issues were resolved. Early motor milestones appeared normal, sitting at 6 months of age and walking at 12 months old. However, expressive speech delay was noted (with first word at approximately 2–2.5 years), which improved dramatically after speech therapy. There were no concerns with receptive speech, and cognitive function appeared normal for age. At 2.5–3 years old, parents noticed gait anomalies, which progressed to frequent falls. On exam at four years, he was unable to walk tandem and had a wide-based gait. He demonstrated abnormal posturing and movements consistent with chorea. No telangiectasias were noted at this time. There was no evidence for cytopenia based on complete blood counts and no evidence for immunodeficiency based on clinical history, immunoglobulin levels, vaccine titers, and clinical flow cytometry for lymphocyte populations. The review of the family history (maternal and paternal; proband is the only child) revealed no history of hyperkinetic movements, chorea, ataxia, pediatric cancer, premature birth, or any birth problems on either side of the family. Elevated serum alpha-fetoprotein (AFP) is a known biomarker for the AT (serum AFP levels are persistently elevated above10 ng/mL in about 95% of AT patients even after the age of two years) (Gatti and Perlman, 1993; Waldmann and McIntire, 1972), but AFP measured at three years and four years were within normal range (3.9 and 4.2 ng/ml, respectively). However, a chromosome analysis of the peripheral blood identified the presence of frequent chromosome translocations t (7; 14) and t (7; 22), which were highly suggestive of AT.

**FIGURE 1:**
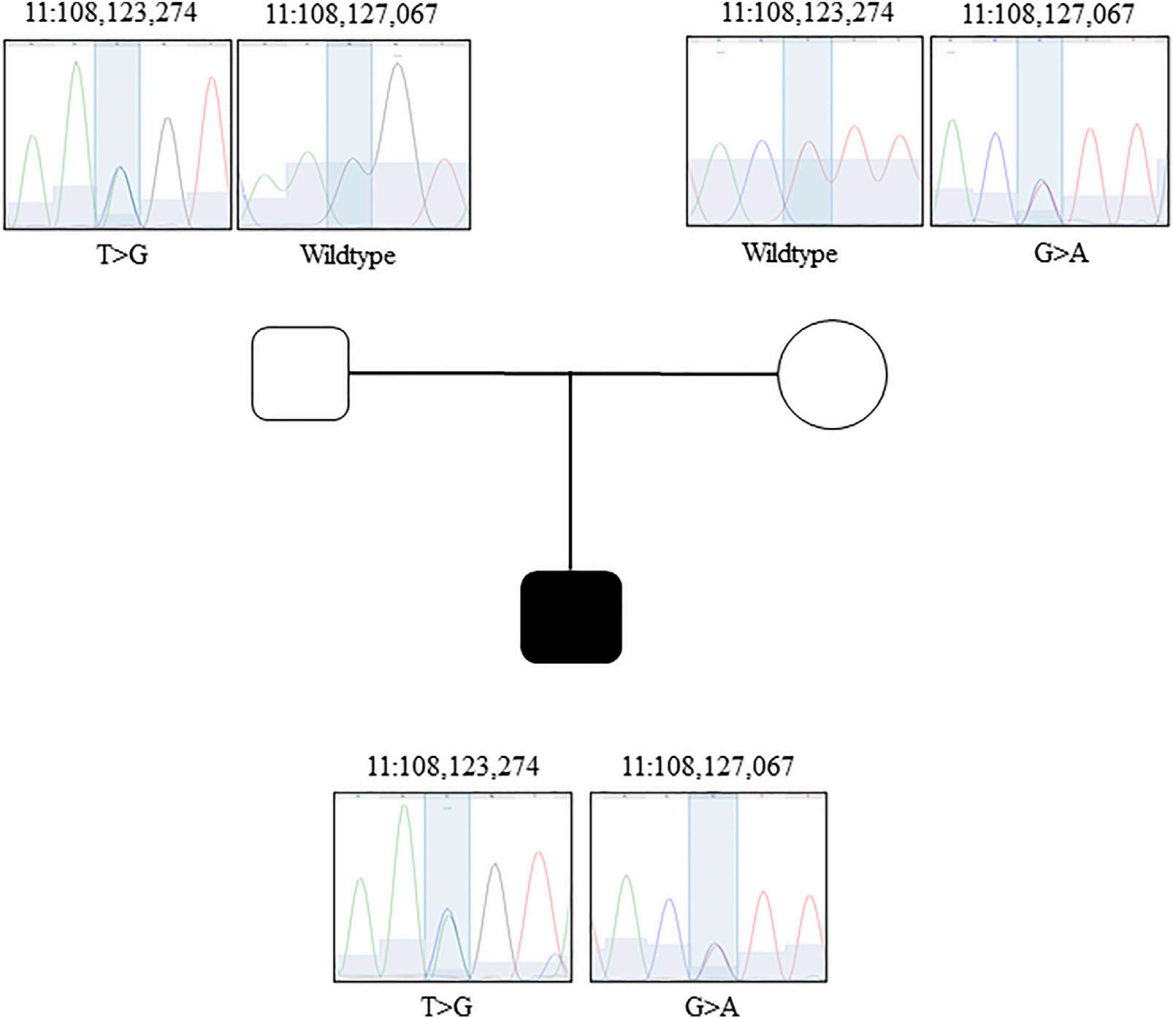
Sanger confirmation of the GS data. Family pedigree and Sanger chromatograms of genomic sequence for each family member (father, mother, proband) for both candidates variants.

The GeneDx clinical ataxia gene panel identified maternally inherited (Figure 1) synonymous variant (NM_000051.3a: c.2250G > A) in the *ATM*. The c.2250G > A variant is rare in the population databases (0.00004387 in gnomAD v2.1.1; 0.00002630 in gnomAD v3.1.1; and 0.000026446 in TopMed database freeze 8) and only present in the heterozygous state. This variant is classified as pathogenic/likely pathogenic in ClinVar (Landrum et al., 2018) by many submitters (∼18) and has been experimentally confirmed to result in exon 14 skipping (Teraoka et al., 1999). However, a second variant, located *in trans*, either inherited from dad or of a *de novo* origin, was not identified. Considering autosomal recessive inheritance of AT, identification of the biallelic loss of function ATM was necessary to molecularly confirm the AT diagnosis.

## Results

### Genome Sequencing

To search for the second *ATM* variant, the family was enrolled in the CombGene research study, approved by the Conjoint Health Research Ethics Board (CHREB) at the University of Calgary (REB19-0245). Written informed consent was obtained for all participants. Using blood-derived DNA, trio genome sequencing (GS)—including proband, unaffected mother, and unaffected father—was performed at The Hospital for Sick Children (SICKKIDS, Toronto, Canada) using the Illumina TruSeq PCR-free DNA library and the Illumina NovaSeq 6000 with a targeted coverage of 40x. The median depth of coverage achieved was 45.1 for proband, 38.3 for mother, and 39.2 for father.

First, we scrutinized the GS data of the *ATM* locus. We looked for rare or novel complex structural variants (Maroilley et al., 2021), mobile element insertions (Tarailo-Graovac et al., 2017a), 5′ and 3′ UTR (Whiffin et al., 2020; Wright et al., 2021; Zhang et al., 2021), deep exonic (Casadei et al., 2019), and deep intronic variants either inherited from father or absent from both parents. These analyses led to the identification of a paternal deep intronic variant NG_009830.1(NM_000051.3): c.1803-270T > G. The variant is absent from all the population databases tested, including our in-house database, gnomAD (v2 and v3) and TopMed database freeze 8. We used three *in silico* tools to probe the predicted effect of this deep intronic variant on splicing with the following results: (1) SpliceAI 0.71 score for acceptor gain at −60 bp and donor gain at −5 bp (Jaganathan et al., 2019); (2) TraP score of 0.24 where 0.232 is 95% and possibly damaging (Gelfman et al., 2017); and (3) CADD-Splice score of 11.35 (Rentzsch et al., 2021).

To be certain that the two variants in the *ATM*, the maternal synonymous and the paternal deep intronic, remained the best potential genetic explanation for this proband, we expanded our GS analyses beyond the *ATM*. Our trio-based genome sequencing analyses, where we consider single nucleotide variants (SNVs), structural variants (SVs) (Maroilley et al., 2021), repeat expansions (Dolzhenko et al., 2020), mitochondrial DNA (Calabrese et al., 2014) according to various modes of inheritance, revealed no additional findings of significance beyond the *ATM* variants.

### Transcript Analysis

**FIGURE 2:**
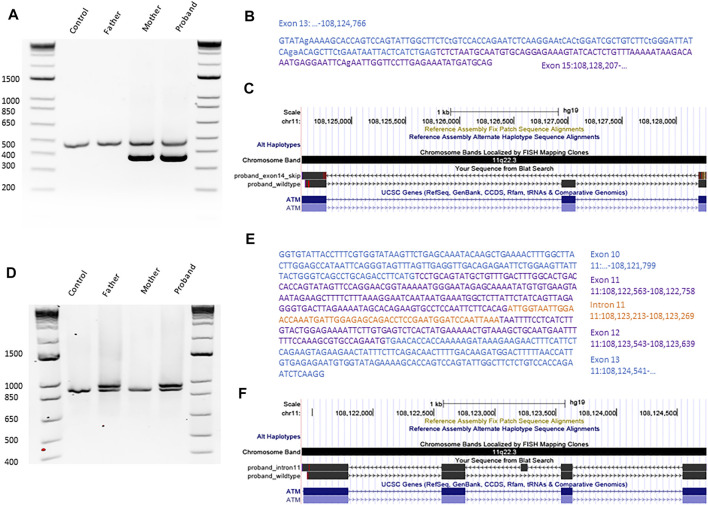
Transcript analyses confirming the impact of both candidate variants on *ATM*. **(A)** PCR gel of RT-PCR products after amplification of cDNA sequences surrounding the variant c.2250G > A for an unrelated control, father, mother, and proband. **(B)** Sequence of the PCR product, shorter than expected from the proband, obtained with Sanger Sequencing. **(C)** Alignment of the proband’s cDNA ATM sequence with ATM RefSeq visualized using UCSC browser. **(D)** PCR gel of RT-PCR products after amplification of cDNA sequences surrounding the variant c.1803-270T > G for an unrelated control, father, mother, and proband. **(E)** Sequence of the PCR product, longer than expected from the proband, obtained with Sanger Sequencing. **(F)** Alignment of the proband’s cDNA ATM sequence with ATM RefSeq in UCSC browser.

Next, we confirmed the *ATM* variants using Sanger sequencing (Figure 1) and established parents as carriers and the proband as compound heterozygous. To investigate the impact of the variants on *ATM* transcript, we performed targeted RNA analyses. The blood from the proband, mother, and father was collected using the PAXgene Blood RNA tubes (BD bioscience) for immediate stabilization of intracellular RNA. Total RNA was manually purified with the PAXgene Blood RNA kit (PreAnalytiX). The RNA was diluted in 10 mM Tris-HCL, pH7.5, with content and purity measured using NanoDROP ONE^c^. The total yield of RNA from each 2.5 ml blood sample was above 3 ug, and the A_260_/A_280_ ratio was within the range of 1.8–2.2. Using the 10 uM of a random hexamer, the cDNA synthesis was performed with Omniscript Reverse Transcription Kit (Qiagen). To probe the transcripts, we performed a PCR on an unrelated healthy control, unaffected father, unaffected mother, and the proband (Figure 2). The primers used to amplify the regions surrounding the c.2250G > A variant are 5′-GCG​TGC​CAG​AAT​GTG​AAC​A-3′ and 5′-GGA​CTC​TTC​TTG​GTA​CAG​TTG​C-3′. The primers designed to detect the impact of the c.1803-270T > G variant are 5′-TTT​TGA​TCT​TGT​GCC​TTG​GCT​AC-3′ and 5′-CTG​AAT​TTG​TAA​TCT​CAG​ATG​AGT​AAT​TAT​TCA​G-3′. The PCR annealing temperature used was 70°C and 67°C, respectively. We used the high fidelity polymerase Phusion (NEB). The PCR products were first checked on 2% agarose gel (Figures 2A,D) to probe any difference in band quantity and size. The gel bands of the PCR fragments were dissected from the gel, purified with GFX™ PCR DNA and Gel Band Purification Kit (Cytiva). All purified PCR products were sent for Sanger sequencing at the University of Calgary DNA Sequencing Facility. We confirmed the exon 14 skipping due to the maternal synonymous variant in both the proband and the mother (Teraoka et al., 1999) (Figures 2A–C). Transcript analyses also revealed that the paternal c.1803-270T > G variant located in intron 11 of the *ATM* results in a larger transcript than expected in both the proband and father (Figures 2D–F). Targeted Sanger sequencing of the product confirmed the aberrant splicing due to the inclusion of 56 base pairs of intron 11 (Figures 2E,F). Our findings for this new deep intronic variant are similar to the findings reported for the other deep intronic variants in the *ATM* (discussed above), where the variant introduces the cryptic splice site and pseudo-exon. *In silico* tests predicted the creation of premature stop codon resulting after translation in a likely non-functional 607 amino-acid ATM protein rather than 3,056 functional ATM protein.

### Functional Analysis

ATM is a critical initiator of the DNA-damage response by the process of phosphorylation of the key substrates involved in several critical cellular processes, such as DNA damage repair, apoptosis, and cell cycle (Phan and Rezaeian, 2021). Recently, the Nationwide Children’s Hospital, Columbus, Ohio, has launched the clinical test DDRFL (DNA Damage Repair Assessment in Lymphocytes in Blood by Flow Cytometry) to assess the non-homologous end joining (NHEJ) DNA double-strand break (DSBs) repair pathway in patients’ lymphocytes (Barmettler et al., 2021). The DDRFL clinical test for further functional assessment with respect to ATM was approved by the local institution for the proband. As part of the test, the phosphorylation of ATM, SMC1, and H2AX is measured after exposure to 2Gy irradiation (at 1 and 24 h) (https://www.nationwidechildrens.org/). Typically, at 1 h after radiation, high expression of these proteins is observed, while at 24 h, the expression returns to normal upon completed DNA DSB repair. However, in our proband, the DDRFL revealed a substantial decrease in phosphorylated pATM (> sevenfold), pSMC1 (> twofold), gamma H2AX (∼threefold) in T cells at 1 h after irradiation with 2Gy. Similarly, the decrease was reported for B cells and NK cells (Supplementary Table S1). Furthermore, the persistence of gamma H2AX 24 h after radiation was indicative of impaired DNA double-strand break repair. These findings are comparable to other patients with the biallelic functional loss of ATM, which supports the pathogenic effect of both variants in our patient, the maternal synonymous and the paternal deep intronic.

## Discussion

Cloning the *ATM* gene in 1995 and establishing that AT is a monogenic disease (Savitsky et al., 1995), despite clinical heterogeneity, had offered the possibility of genetic diagnosis for patients with clinical features of AT (Savitsky et al., 1995). Furthermore, it allowed prenatal testing for the families and identification and subsequent screening of unaffected heterozygous carriers with a higher predisposition to cancer. However, similar to other rare diseases, patients with AT still face diagnostic delays and the “missing heritability” problem (Maroilley and Tarailo-Graovac, 2019), where a genetic cause is difficult to establish in some patients despite a suggestive clinical AT phenotype (Schon et al., 2019). Previously, our work has led to the identification of potential sources of missing heritability (Maroilley and Tarailo-Graovac, 2019) in rare disease patients, such as mobile element insertions (Tarailo-Graovac et al., 2017a), complex genome rearrangements (van Kuilenburg et al., 2018), somatic mosaicisms (Matthews et al., 2017; Ragotte et al., 2017), repeat expansion (van Kuilenburg et al., 2019), and variable penetrance and expressivity (Tarailo-Graovac et al., 2017b). Herein, we report on the unanticipated finding of compound heterozygosity for a synonymous and a deep intronic variant. We then further highlight the power of genome sequencing in reducing missing heritability when diagnosing rare disease patients by enabling consideration of more complex genetic mechanisms and exploring both coding and non-coding regions.

Identifying variants from non-coding regions using the genome sequencing data and interpreting their pathogenicity is challenging. In our experience, the patients with well-defined clinical features and highly suspected clinical diagnoses (e.g., AT), yet with no or partial genetic diagnosis, are important in facilitating the discovery of unexplored genetic mechanisms in human genetic disease and hence the understanding of the functional elements in the human genome. Importantly, the discovery of new disease-causing mechanisms can improve available clinical tests and future diagnostics and screening. For our patient, even though a clinical diagnosis of AT was highly suspected, identification of the second variant confirmed the diagnosis for the child, which has implications for clinical surveillance (respiratory and malignant disease) (Bhatt et al., 2015), and therapy (particularly for management of immune deficiency, e.g., with IVIg), which improves outcomes (Nowak-Wegrzyn et al., 2004; Rothblum-Oviatt et al., 2016).

Diagnosis of AT also has significant clinical implications for detecting carriers at risk for cancer predisposition and require preventative surveillance and management (van Os et al., 2016). As explained by Savitsky et al., 1995, it is only once we know the full spectrum of the AT disease-causing variants that we will be able to effectively screen for carriers who are predisposed to cancer and implement precise preventative surveillance and management protocols to save lives (Savitsky et al., 1995). Various clinical tests, albeit focused on protein-coding regions of the gene, do include reported disease-causing variants located in non-coding regions. The discovery of the c.1803-270T > G variant as disease causing in our patient may in future also help diagnose other AT patients or identify carriers that may be at higher risk of cancer.

Finally, identification of full diagnosis even where clinical diagnosis is highly suspected is crucial for accurate genetic counseling and prenatal diagnosis for the family, and more precise clinical management, as well as clinical trials and identification of treatment avenues. Genetic mechanisms are increasingly being leveraged to develop novel therapeutics, for example, by targeting control of alternative splicing to compensate for the affected allele. One example is the recent report on complete molecular diagnosis in highly suspected Batten’s disease and the development of the milasen, patient-customized splice-modulating antisense oligonucleotide therapy (Kim et al., 2019). Deep intronic variants, in particular, may be amenable to design of antisense oligonucleotide drugs that could help mask the cryptic splice sites generated by the deep intronic variants, skip the pseudo-exon, and restore the transcripts (Sangermano et al., 2019; Siva et al., 2014). The *ATM* gene has already been explored in the antisense oligonucleotide drugs research (Cavalieri et al., 2013; Du et al., 2007).

## Data Availability

The datasets for this article are not publicly available due to concerns regarding participant/patient anonymity. Requests to access the datasets should be directed to the corresponding authors.
