# Supplementary material for: Case Report: Biallelic Loss of Function ATM due to Pathogenic Synonymous and Novel Deep Intronic Variant c.1803-270T > G Identified by Genome Sequencing in a Child With Ataxia–Telangiectasia
Source: Front Genet. 2022 Jan 25;13:815210. doi: 10.3389/fgene.2022.815210 (PMC8822238; doi:10.3389/fgene.2022.815210)
Supplement: Supplementary file 1 [file Table1.DOCX]

**Supplemental Information**

Table S1: Functional analyses of ATM protein by the DDRFL (Nationwide Children's).

| Lymphocyte subsets | DNA repair protein phosphorylation | | Unit | 1h  post-IR^1^ | Ref. Interval for 1h result | 24h post-IR^1^ | Ref. Interval for 24h result |
| --- | --- | --- | --- | --- | --- | --- | --- |
| CD3+ T-cells | pATM^2^ | | Delta (%)^4^ | 59.5 | 91.2-98.2 |  |  |
|  |  |  | Ratio (MFI)^5^ | 2.9 | 2.8-8.7 | 0.9 | 0.9-2.7 |
|  | pSMC1^3^ | | Delta (%)^4^ | 82.3 | 90.2-97.7 |  |  |
|  |  |  | Ratio (MFI)^5^ | 3.1 | 2.4-4.9 | 2.3 | 1.5-2.9 |
|  | γH2AX | | Delta (%)^4^ | 96.2 | 92.3-98.4 |  |  |
|  |  |  | Ratio (MFI)^5^ | 44.1 | 19.4-74.6 | 4.8 | 0.8-2.4 |
|  | 24h post-IR^1^ | % Apoptotic | Fold-increase |  |  | 2.2 | 3.2-26.6 |
|  |  | % Dead | Fold-increase |  |  | 3.5 | 1.4-30.2 |
| CD19+ B-cells | pATM^2^ | | Delta (%)^4^ | 20.5 | 67.7-93.5 |  |  |
|  |  |  | Ratio (MFI)^5^ | 3.0 | 1.3-5.1 | 0.9 | 0.7-2.4 |
|  | pSMC1^3^ | | Delta (%)^4^ | 78.8 | 79.7-96.2 |  |  |
|  |  |  | Ratio (MFI)^5^ | 4.6 | 2.5-6.4 | 2.5 | 1.4-3.8 |
|  | γH2AX | | Delta (%)^4^ | 83.0 | 75.9-96.5 |  |  |
|  |  |  | Ratio (MFI)^5^ | 89.2 | 26.1-411.7 | 8.0 | 0.6-6.4 |
|  | 24h post-IR^1^ | % Apoptotic | Fold-increase |  |  | 1.2 | 0.9-18.2 |
|  |  | % Dead | Fold-increase |  |  | 2.0 | 1.2-22.7 |
| CD335+ (NKp46) NK-cells | pATM^2^ | | Delta (%)^4^ | 66.2 | 95.8-99.3 |  |  |
|  |  |  | Ratio (MFI)^5^ | 4.0 | 3.2-12.7 | .DNR | 1.0-2.4 |
|  | pSMC1^3^ | | Delta (%)^4^ | 78.4 | 90.9-97.7 |  |  |
|  |  |  | Ratio (MFI)^5^ | 2.6 | 2.3-4.0 | .DNR | 1.2-2.2 |
|  | γH2AX | | Delta (%)^4^ | 93.6 | 95.7-99.6 |  |  |
|  |  |  | Ratio (MFI)^5^ | 39.0 | 17.6-98.7 | .DNR | 0.9-3.1 |
|  | 24h post-IR^1^ | % Apoptotic | Fold-increase |  |  | .DNR | 5.5-32.5 |
|  |  | % Dead | Fold-increase |  |  | .DNR | 8-41.6 |

^1^ IR = irradiation (IR)

^2^ pATM = phospho-ATM

^3^ pSMC1 = phospho-SMC1

^4^ Delta (%) = % of cells expressing three proteins of interest

^5^ MFI ratio = the ratio of irradiated cells compared to radiated cells during the assay.
